# Robotic Proximal Ureteropyelostomy After Unsuccessful Endourologic Management of Complicated Proximal Ureteral Stone Disease

**DOI:** 10.1089/cren.2015.29012.dop

**Published:** 2015-10-01

**Authors:** Daniel Olvera-Posada, Marie Dion, Husain Alenezi, Hassan Razvi, Stephen E. Pautler

**Affiliations:** ^1^ Division of Urology, Department of Surgery, Schulich School of Medicine & Dentistry, Western University, London, Ontario, Canada.; ^2^ Divisions of Urology and Surgical Oncology, Departments of Surgery and Oncology, Schulich School of Medicine & Dentistry, Western University, London, Ontario, Canada.

## Abstract

We present a clinical case of a 66-year-old female with a left ureteropelvic junction impacted renal calculi associated with a tortuous ureter. After a failed combined retrograde and antegrade endoscopic procedure, a robot-assisted laparoscopic ureteropyelostomy was successful.

## Clinical History

A 66-year-old female with a history of recurrent nephrolithiasis was referred for the presence of right proximal ureteral calculi. She had previously been treated with ureteroscopy and laser lithotripsy for right renal calculi and most recently had passed a 3 mm stone 8 months ago. She had no complaints of recent flank pain, gross hematuria, or lower urinary tract symptoms. She had been treated for recurrent infections by her general practitioner every 3 months for the last 2 years.

The patient's medical history was significant for melanoma, type 2 diabetes, hypertension, and hypercholesterolemia. She had several previous surgeries, including bilateral knee replacements, three cesarean sections, a melanoma excision from her left shoulder, and the aforementioned ureteroscopy. She was a previous smoker for 20 years and consumed minimal alcohol. She retired from a marketing position and has five children.

## Diagnosis

**FIG. 1.**
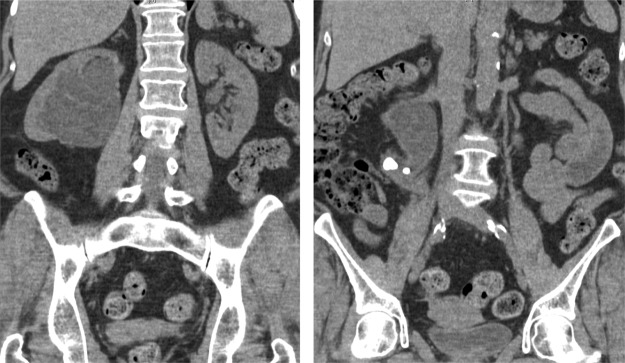
Preoperative computed tomography scan, coronal planes showing marked hydronephrosis associated with the presence of two calculi and in the ureteropelvic junction (UPJ) and an extremely tortuous ureter.

A renal ultrasound showed right-sided hydronephrosis and two large stones in the right proximal ureter. A CT scan confirmed the presence of two large stones measuring 14 × 7 and 7 × 5 mm in the proximal ureter with severe inflammation of the surrounding tissue, severe hydronephrosis, and marked tortuosity of the ureter (Fig. 1).

## Intervention

Ureteroscopy with possible percutaneous approach if necessary was planned based on the anticipated difficulty of endoscopic access to the calculi. Intraoperative retrograde contrast injections showed a significant S-shape curvature of the ureter. Despite passage of a guidewire, persistent ureteral tortuosity made it difficult to approach the stone in either antegrade or retrograde direction.

During follow-up, a renogram was obtained to determine whether the right kidney still contributed 37% of the overall renal function. The patient agreed to undergo a robot-assisted laparoscopic ureteral resection and reconstruction.

**FIG. 2.**
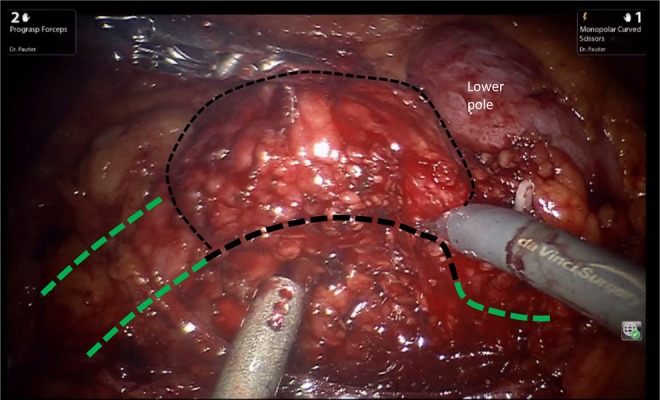
Intraoperative appearance showing the inflammatory mass in the UPJ area, below the *lower pole* of the right kidney. The healthy ureter is shown at the left aspect of the suction device, *green lines* show the healthy ureter and pelvis, and *black dotted lines* depict the excised area.

**FIG. 3.**
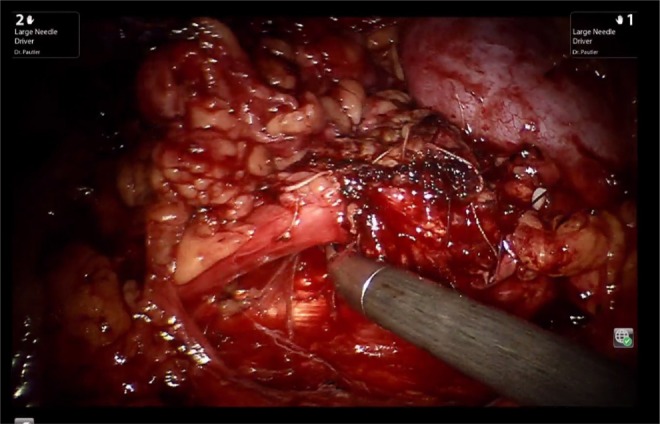
The area of reconstruction is shown, following the surgical principle of a water-tight, tension-free anastomosis.

Using the Hasson technique in the right midclavicular line, the camera port was placed. Two additional 8-mm ports for the robotic arms were triangulated and the assistant port was positioned lateral to the umbilicus. After visual inspection of the abdomen, the line of Toldt was medially reflected and Gerota's fascia was opened. A large inflammatory mass inferior and adherent to the kidney's lower pole was visualized (Fig. 2). With meticulous dissection, the pelvis and proximal ureter were identified and isolated. The ureteropelvic junction (UPJ) and the region of greatest inflammation around the stone were identified and the anterior aspect of the pelvis was opened. This incision was carried out along the length of the ureter inferiorly until the region of stone impaction was passed and normal tissue identified. The ureter was then divided inferior to the stone and inflammatory process. The UPJ was divided superior to the stone. The distal ureter was dilated sufficiently so that it did not require spatulation and there was sufficient length to anastomose the ureter to the renal pelvis. Interrupted vicryl sutures were used to complete the tension-free anastomosis (Fig. 3). A Double-J stent was placed to allow the anastomosis to heal for 5 weeks. The operative time was 218 minutes. The patient was discharged on the third postoperative day and had an unremarkable recovery.

## Follow-Up and Outcomes

**FIG. 4.**
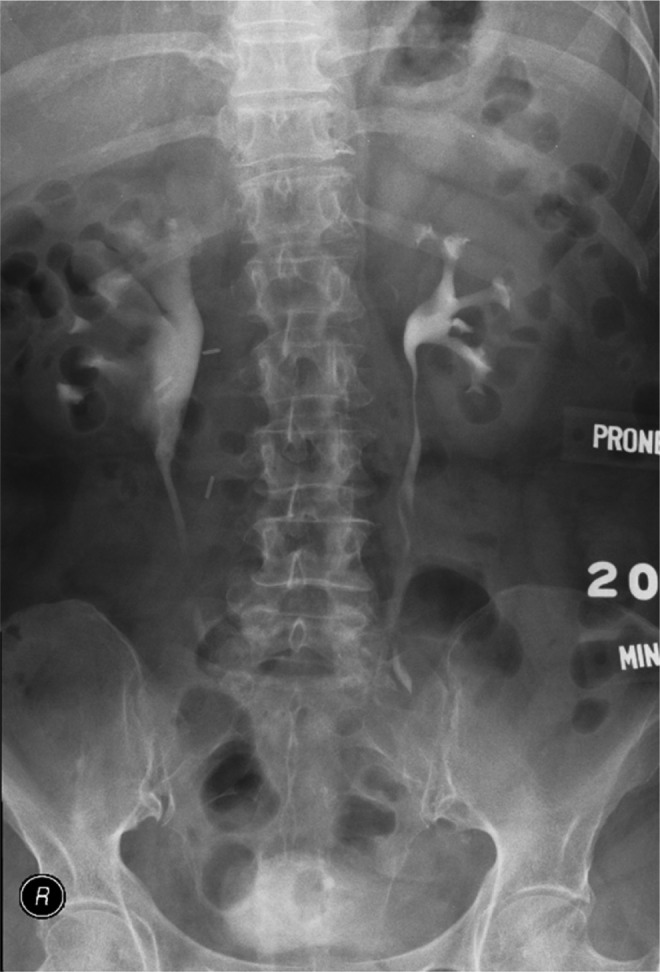
Postoperative intravenous pyelogram showing adequate patency of the right UPJ with mild hydronephrosis, probably residual after the longstanding obstruction.

An intravenous urogram 6 weeks after surgery showed mild pyelectasis but good drainage (Fig. 4). A renogram at 1 year showed increased uptake in the right kidney at 42% and a diuretic half-life of 14 minutes. The patient had remained well with no urinary tract infections or flank discomfort. Resected tissue was negative for malignancy.

## Discussion

Previous authors have demonstrated the safety and efficacy of a robotic approach for complicated reconstruction of the upper urinary tract. In a series of 65 renal surgeries, including pyeloplasty with stone extraction and other ureteral reconstructive procedures, Mufarrij et al. report high radiographic and patient symptom resolution rates of 97.3% and 100.0%, respectively, with extremely low complication rates. They define success as a diuretic half-life of less than 20 minutes or resolution of hydronephrosis and prompt excretion of contrast. In this series, they attribute their success to adherence to the key principles of reconstruction, including minimizing manipulation, spatulation, tension relief with mobilization where necessary, and stent and drain placement. They also comment that the robotic approach was helpful in situations where previous surgery had occurred or there was retroperitoneal fibrosis due to their improved ability to handle tissues extremely delicately and increased maneuverability and visualization.^1^ Atug et al. concluded that pyeloplasty with concomitant stone extraction was feasible with a robotic approach and only increases the operative time.^2^ Another series of 29 patients undergoing robotic repair of complicated UPJ obstruction demonstrates successful outcomes after complicated UPJ obstruction with multiple stones.^3^ We would agree that in this case, the robotic approach was helpful in dissection of the large inflammatory mass surrounding the stone and proximal ureter.

This case exemplifies that a robotic approach is a feasible and good option after an unsuccessful antegrade and retrograde approach. Furthermore, despite the appearance of parenchymal thinning on the patient's initial CT scan 1 year after treatment, her affected kidney appeared to be contributing about 42% function.

## Abbreviations Used

CT: computed tomography
IVP: intravenous pyelogram
UPJ: ureteropelvic junction
